# Antibacterial effects of silver diamine fluoride on multi-species cariogenic biofilm on caries

**DOI:** 10.1186/1476-0711-12-4

**Published:** 2013-02-26

**Authors:** May Lei Mei, Quan-li Li, Chun-Hung Chu, Edward Chin-Man Lo, Lakshman Perera Samaranayake

**Affiliations:** 1 Faculty of Dentistry, The University of Hong Kong, Pok Fu Lam Rd, Hong Kong, People’s Republic of China; 2 Stomatology Collage, Anhui Medical Univerisity, Hefei, People’s Republic of China

## Abstract

**Backgrounds:**

Silver diamine fluoride (SDF) has clinical success in arresting dentin caries, this study aimed to investigate its mechanism of action.

**Methods:**

Using a computer-controlled artificial mouth, we studied the effect of 38% SDF on cariogenic biofilms and dentin carious lesions. We used five common cariogenic bacteria (*Streptococcus mutans, Streptococcus sobrinus, Lactobacillus acidophilus, Lactobacillus rhamnosus and Actinomyces naeslundii*) to form a cariogenic biofilm that generated carious lesions with a depth of approximately 70 um on human dentin blocks. We applied 38% SDF to the lesions in the test group and water to those in the control group. The blocks were incubated in the artificial mouth for 21 days before evaluation. Microbial kinetics, architecture, viability and distribution were evaluated every 7 days using colony forming unit (CFU), scanning electron microscopy and confocal laser scanning microscopy. The physical properties of the carious lesions were evaluated with microhardness testing, energy dispersive spectroscopy (EDS) and Fourier transform infra-red spectroscopy (FTIR).

**Results:**

The CFU results revealed fewer colony forming units in the test group compared with the control group (p < 0.01). Scanning electron microscopy and confocal microscopy showed less bacterial growth in the test group, and confluent cariogenic biofilm in the control group (p < 0.01). The microhardness and weight percentages of calcium and phosphorus in the test group from the outermost 50mum were higher than in the control group (p < 0.05). EDS showed that calcium and phosphous were higher in outer 50 mum in test groups than in the control FTIR revealed less exposed collagen I in the test lesions compared with the control group (p < 0.01).

**Conclusions:**

38% SDF inhibits multi-species cariogenic biofilm formation on dentin carious lesions and reduces the demineralization process.

## Background

The topical application of silver diamine fluoride (SDF) is a cost-effective agent in the management of caries [1]. Clinical studies have shown that SDF is effective in arresting dentin caries [2-4] and enamel caries [5], but its mechanism of action remains unclear. Laboratory studies have reported that SDF prevents the formation of *Streptococcus mutans* or *Actinomyces naeslundii* mono-species biofilms [6]. In addition, SDF inhibits the demineralization of dentin [6,7]. SDF is commonly used at 38% [8]. A lower concentration at 12% is also available but clinical studies found that it was not as effective as 38% in arresting dental caries among children [9,10].

Caries is a polymicrobial infection process [11] in the complex oral cavity that varies greatly with intraoral location over time and among individuals [12]. Therefore, some researchers suggest studying caries in a sophisticated artificial mouth model because it allows biofilm to grow within a controlled environment that mimics the *in vivo* oral niches and habitats of the human mouth [13]. Artificial mouth model has been adopted in different researches, such as biodiversity of dental plaque [14] and antimicrobial effect of chlorhexidine [15] and generating consortia of bacterial biofilm [12]. The aim of this study was to investigate the effects of 38% SDF on multi-species biofilms developed from five common cariogenic bacteria on dentin carious lesions in a computer-controlled artificial mouth. We took microbial, chemical, and physical measurements to gain an inclusive understanding of the effects of SDF on dentin carious lesions infected by cariogenic biofilm.

## Materials

### Specimen and artificial mouth preparation

This study was approved by the Institutional Review Board (IRB UW08-052). Seventy-two dentin blocks (2 × 2 × 4mm^3^) were prepared from sound human third molars. Half of the blocks’ surfaces were coated with acid-resistant nail varnish to serve as an internal control. Twelve of the blocks were embedded in an acrylic resin disk designed to fit inside a computer-controlled artificial mouth [12], and then sterilized for 16 hours with ethylene oxide (Amsco Eagle 2017 EO sterilizer, Mentor, OH, USA). Ethylene oxide is a common method for sterilizing dental hard tissue for in vitro studies [16].

Five common species of cariogenic bacteria—*Streptococcus mutans* ATCC (American Type Culture Collection) 35668*, Streptococcus sobrinus* ATCC 33478, *Lactobacillus acidophilus* ATCC 9224*, Lactobacillus rhamnosus* ATCC 10863, and *Actinomyces naeslundii* ATCC 12014—were selected for the study. Five equal aliquots of 10 ^7^ CFU/mL of each organism were mixed and inoculated on the dentin blocks. The blocks were then incubated anaerobically in a brain-heart infusion broth at 37°C for 3 days to create carious lesions approximately 70 μm in depth on the exposed surfaces of the dentin blocks. Half of the blocks were assigned as a test group, and the other half were used as a control. In the test group, 38% SDF solution (Saforide, Toyo Seiyaku Kasei Co. Ltd., Osaka, Japan) was applied topically to the exposed surfaces of the blocks with a gravimetric micro-brush. The average amount of SDF applied was 0.22 mg (8.8 μg fluoride). The blocks in the control group were treated with sterile distilled water.

All of the blocks were then incubated in the artificial mouth, which provided a controlled environment for the growth of the biofilms. A humidified gas mixture of 5% carbon dioxide and 95% nitrogen was supplied continuously at 60 ml/min. The temperature inside the artificial mouth was maintained at 37°C. Simulated oral fluid (defined medium mucin) was continuously supplied at 2.5 mL/hr to mimic salivary flow. Sucrose solution at 5% was supplied for 6 minutes every 8 hours with a flow rate of 15 mL/hr. The conditions were monitored by a computer program (LabVIEW® software Version2.2). Twenty-four blocks (12 treated with SDF and 12 treated with water) were removed for evaluation after 7 days. The 7-day period allowed the bacteria to grow and mature into biofilm [17]. Another 24 blocks were assessed after 14 days, and the remaining 24 blocks were studied after 21 days.

### Biofilm assessment

To determine the total bacterial colony forming unit (CFU), serial 10-fold dilutions in phosphate buffered solution of homogenized plaque were plated in duplicate using a spiral plater (Autoplate 4000, Spiral Biotech Inc., Norwood, MA). Horse blood agar (Defib Horse Blood, Hemostat laboratories, Dixon, USA) was used to determine CFU of cultivable species. Selective media plates were used for specific identification of individual species. Mitis Salivarius, Rogosa and Actinomyces agar plate were used for Mutans Streptococci, Lactobaciili and Actinomycetes identification, respectively. The plates were incubated for 72 hours anaerobically before CFU counting. Isolated colonies were confirmed by Gram stain and catalase test. The CFU counting results were re-checked by a technician who do not know the experiment design.

The surface topographies of the biofilms were studied under scanning electron microsocpy (SEM) (Leo 1530, LEO, Oberkochen, Germany) at 12 kV in high-vacuum mode [18]. Confocal laser scanning microscopy (CLSM) was used to study the viability and distribution of the biofilms. The bacteria on the dentin surfaces were labeled *in situ* using two fluorescent probes: propodium iodide and SYTO-9 dye (LIVE/DEAD BacLight Bacterial viability kit, Molecular Probes, Eugene, OR, USA). Ten cellular images from the middle layer of each biofilm were obtained using CLSM (Fluoview FV 1000, Olympus, Tokyo, Japan) and analyzed using an image analysis software (Image J, National Institutes of Health, Bethesda, MD, USA). The red-to-green ratio was calculated to denote the ratio of dead-to-live bacteria, respectively. A high ratio indicates the positive anti-microbial effect of the studied therapeutic agent [6].

### Dentin blocks assessment

Each dentin block was sectioned vertically, midway across the caries, or the surface was demineralized after biofilm collection. The hardness of the carious lesions was evaluated through Knoop microhardness testing (Leiz Microhardness Tester; Ernst Leitz Wetzlar GmbH, Wetzlar, Germany). The Knoop indenter was applied to the carious lesions for 10s each with a force of 98 × 10^-3^N. The microhardness was determined at 25 μm below the surface of the dentin blocks in increments of 25 μm on both exposed (experimental) and varnished (internal control) sites. Five sets of Knoop hardness number (KHN) measurements were made on each block, on parallel tracks approximately 150 μm apart. Relative microhardness was calculated from the measured KHN on the carious dentin, divided by the KHN on sound dentin [19].

The mineral content of the carious lesions in terms of elemental calcium (Ca), phosphorus (P), and the Ca/P ratio was assessed by energy dispersive spectroscopy (EDS) (Model 7426; Oxford Instruments, Oxford, UK) under SEM. The points used for elemental analysis were chosen in a region close to the microhardness indentations, and therefore the interval between successive points was also 25 μm.

The potential changes in the organic structure of the carious lesions were analyzed by Bio-Rad Fourier transform infrared spectroscopy (FTIR) (UMA 500, Bio-Rad Laboratories, Hercules, CA, USA) and calculated from the spectrally derived matrix-to-mineral ratio (the ratio of the integrated area of protein amide I absorbance from 1585 to 1720 cm^-1^ to that of phosphate [HPO_4_^2-^] absorbance from 900 to 1200 cm^-1^). The spectra for the carious lesions were obtained through the average acquisition of data at the spatial resolution achieved with a 100 × 100 μm^2^ aperture over the lesions surfaces. The log value of the [amide I: HPO_4_^2-^] absorbance ratio was used as an indicator of the extent of dentin demineralization due to the carious activity of the biofilm.

### Statistical analysis

All of the data were assessed for a normal distribution using the Shapiro-Wilk test for normality (p > 0.05). The parametric *t* test was used to compare log CFU, relative microhardness, weight percentage of Ca and P, Ca/P ratio, and log [amide I: HPO_4_^2-^] ratio between the test and control groups. All of the analyses were conducted using SPSS version 19 software (SPSS Inc., Chicago, Illinois, USA). A cutoff level of significance of 5% was chosen for all of the statistical analyses.

## Results

### Biofilm assessment

The growth kinetics (CFU) of total bacteria, Mutans Streptococci, Lactobacilli, and Actinomycetes in the test and control groups are shown in Table 1. The results of the total log CFU reveal that SDF inhibited the growth of all five bacteria on the carious dentin by the end of day 7, day 14, and day 21 (p < 0.01). At the end of day 7, the CFU in the test group dropped to low values (log CFU < 1) for all of the species. Although the log CFU increased over time, the values were significantly lower than those of the control group.

**Table 1:** Bacterial count (log CFUs) and red/green in test (SDF-treated) and control (n=10)

		**Log CFUs**				**Red/Green (CLSM)**
		**Total bacteria**	**Mutans streptococci**	**Lactobacilli**	***A.naeslundii***	
Day 7	Test	0.65±0.71	0.27±0.65	0.43±0.37	0.83±0.91	2.19±0.55
	Control	8.66±0.30	5.63±0.87	7.04±0.28	6.75±0.19	0.22±0.08
	P value	<0.01	<0.01	<0.01	<0.01	<0.01
Day 14	Test	4.94±0.07	3.99±0.08	4.00±0.04	4.30±0.44	1.64±0.54
	Control	10.29±0.38	7.71±0.08	9.56±0.51	8.80±0.12	0.06±0.02
	P value	<0.01	<0.01	<0.01	<0.01	<0.01
Day 21	Test	5.72±0.19	4.39±0.42	5.42±0.17	5.26±0.65	0.92±0.36
	Control	12.20±0.84	8.11±0.11	12.69±0.39	10.42±0.29	0.004±0.001
	P value	<0.01	<0.01	<0.01	<0.01	<0.01

**Figure 1:**
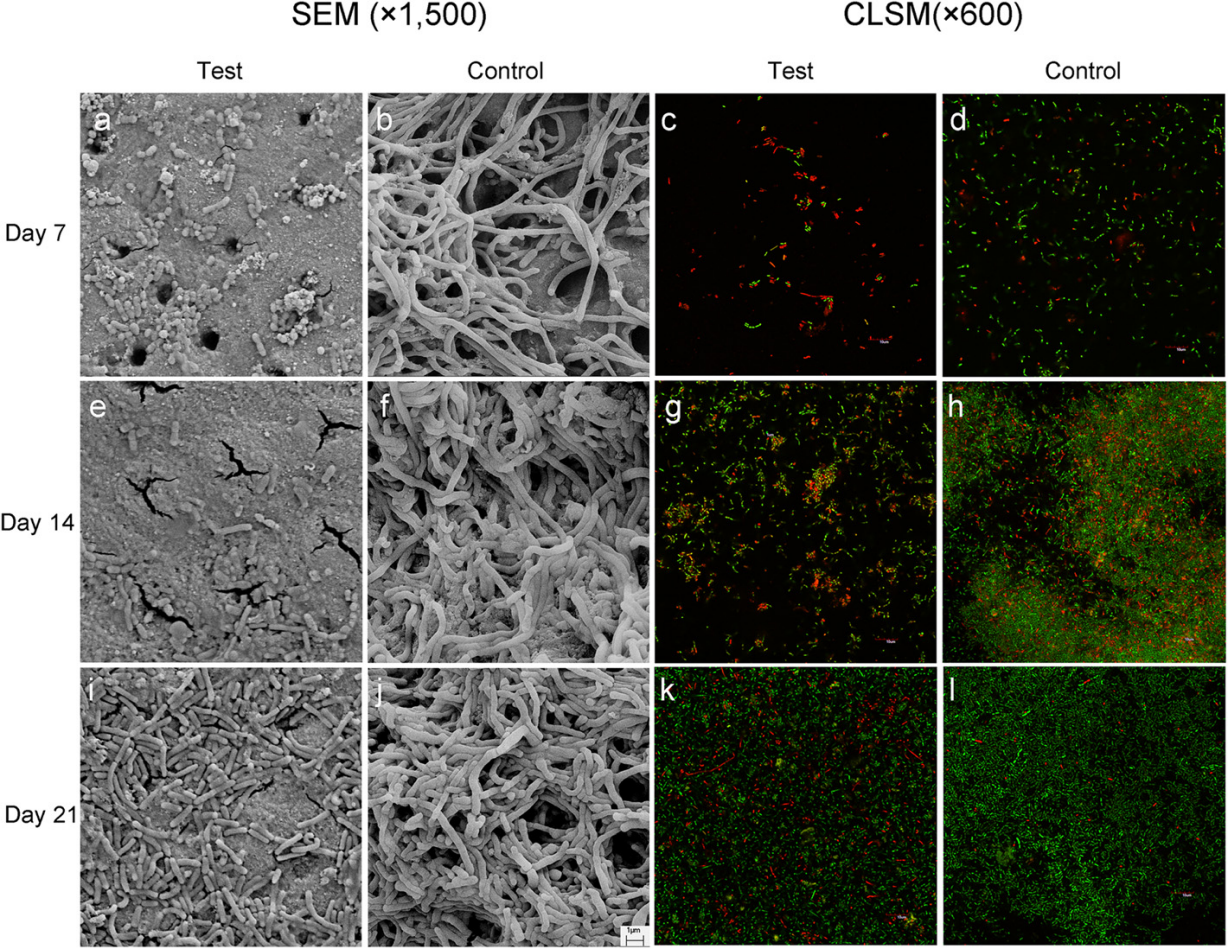
SEM and CLSM images of test (SDF-treated) and control at days 7, 14, and 21.

Bacteria could rarely be detected on the dentin surfaces in the test group (Figure 1a). Moreover, the dentinal tubule orifices were smaller in the test group than those in the control group (Figure 1b). Aggregates of bacteria were observed on the dentin surfaces by day 14 (Figure 1e); and there were partially occluded dentinal tubule orifices. A mono-layer of biofilm was found to cover the majority of the dentin block surfaces and the dentinal tubule orifices were occluded on day 21 (Figure 1i). The biofilm in the control group was confluent (Figure 1f and 1j). Three-dimensional CLSM images suggested a mixture of bacteria arranged in a complex, multi-layered structure. According to the morphology of the bacteria in the CLSM images, Lactobacilli dominated the upper layers (away from the substratum) of biofilm, Mutans Streptococci were commonly found in the lower layers (near the substratum), and more *A. naeslundii* were found in the transitional middle layers (data not shown). The red-to-green ratio, calculated from CLSM images, showed significantly higher ratios in the test group than in the control groups at the ends of days 7, 14, and 21 (Figure 1c,d,g,h,k,l and Table 1).

### Dentin blocks assessment

**Figure 2:**
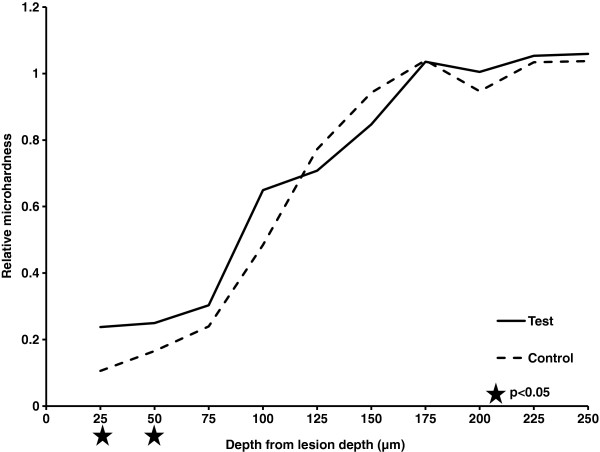
Relative microhardness of test (SDF-treated) and control lesions according to lesion depth (n = 10).

Figure 2 records the relative microhardness of dentin in the test and control groups, according to the distances from the lesions surfaces. The relative microhardness at 25 to 50 μm below the surface was higher in the test group than in the control group (p < 0.05). Elemental analyses revealed that the calcium and phosphorus weight percentages in the test group were also higher than those in the control group in the outer 25 and 50 μm (Tables 2). The dentin surfaces treated with SDF had significantly lower log [amide I: HPO_4_^2-^] than the control group at the ends of days 7, 14, and 21. The logs [amide I: HPO_4_^2-^] for the test and control groups were 0.23 ± 0.04 and 0.47 ± 0.06 (p < 0.01) at day 7, 0.22 ± 0.06 and 0.84 ± 0.36 (p < 0.01) at day 14, 0.22 ± 0.11, and 1.18 ± 0.19 (p < 0.01) at day 21, respectively.

**Table 2:** Relative Ca and P weight% and Ca/P ratio of test (SDF-treated) and control lesions according to lesion depth (n=10)

**μm**	**Ca**			**P**			**Ca/P**		
	**Test**	**Control**	**p**	**Test**	**Control**	**p**	**Test**	**Control**	**p**
25	14.82±3.35	5.02±3.52	**0.01**	6.94±1.95	2.45±1.85	**0.01**	2.16±0.19	2.08±0.29	0.76
50	22.96±4.78	14.66±5.75	**0.04**	10.73±1.41	7.13±2.99	**0.04**	2.13±0.24	2.09±0.24	0.57
75	23.54±3.30	22.85±5.69	0.68	14.44±2.60	11.33±3.34	0.16	2.03±0.12	2.05±0.15	0.12
100	24.65±3.07	26.08±2.84	0.61	14.76±3.17	13.26±1.81	0.22	2.05±0.21	1.98±0.10	0.50
125	24.34±3.28	22.63±1.47	0.24	12.86±1.30	13.74±0.53	0.25	1.91±0.33	1.94±0.10	0.21
150	23.06±4.80	22.67±2.89	0.57	13.04±2.67	9.79±1.79	0.14	2.09±0.16	1.91±0.11	0.12
175	25.32±7.39	20.69±5.59	0.17	12.04±2.12	9.73±1.21	0.11	2.17±0.33	1.95±0.05	0.18
200	23.59±10.45	24.32±5.40	0.33	13.89±4.14	12.01±5.28	0.51	2.14±0.19	2.16±0.34	0.94
225	24.87±3.94	23.72±7.71	0.43	12.67±2.78	13.32±3.17	0.63	1.92±0.15	2.09±0.26	0.20
250	25.79±9.49	27.63±2.85	0.63	14.97±1.31	14.07±1.24	0.36	2.01±0.07	1.96±0.06	0.52

## Discussion

The computer-controlled artificial mouth used in this study is an advanced model system used for caries research [15]. The model mimics real mouth conditions in terms of temperature, humidity, sucrose supply, and saliva flow rate. This artificial mouth model could provide useful information to predict clinical outcome. Based on the findings we got from this study, subsequent study could be performed to assess the extent of the data extrapolation. Nevertheless, the artificial mouth model system creates its own stable environment that differs from *in vivo* situations. The limitations of such an in vitro study must be taken into account when considering the findings of this study. It is noteworthy that dental caries is a polymicrobial infection process, and the mouth had over 700 species of oral bacteria [10]. Five common cariogenic bacteria have been used to form cariogenic biofilm in this study. It is however still far from and is incomparable with the natural biofilm causing caries. The results cannot be extrapolated directly to the *in vivo* situation, and caution should be exercised in their interpretation.

The early stage of bacterial invasion in the caries process involves Streptococci, Lactobacilli, and Actinomycetes [20]. *Streptococci mutans* and *Streptococci sobrinus* are two of the most important odontopathogens involved in the initiation and progression of caries [21]. Caries can be artificially induced in vitro through inoculation onto tooth enamel. *Lactobacilli rhamnosus* and *Lactobacilli acidophilus* are the two most abundant species frequently found in both superficial and deep carious lesions, where the pH tends to be acidic [7]. *Actinomycetes naeslundii* has the potential to invade dentinal tubules and is associated with root caries [22]. For the foregoing reasons, these five cariogenic species from the three specific genera were chosen to form a multi-species cariogenic biofilm.

SDF has been used to manage caries in particular in patients with high caries prevalence [23]. It’s simplicity of application enables it’s use on young children [2]. The inherent disadvantage of using SDF to arrest caries is that the lesions will be stained black. It has been suggested that when carious dentin is treated with SDF, silver phosphate is formed and precipitated [23]. 38% SDF is a colorless solution containing 44,800 ppm fluoride ions. Its high fluoride concentration can be toxic when swallowed in large doses [23].

This study demonstrated SDF-inhibited cariogenic biofilm formation. The inhibition was obvious in the first 7 days after SDF application based on a very low CFU counting, and the CFU increased over time. Since the amount of bacteria in the biofilm after 7 days was small, the variation between samples was relatively large (Table 1). In this study, computer software was used to differentiate different color and area to make quantitative analysis of CLSM [24]. The results however might vary due to uneven distribution of the amount bacteria in different thickness of the biofilm. Furthermore, the quality of images could be affected by conditions such as brightness, white balance and contrast. Therefore, findings of the CLSM were used support the conclusion.

Both the CFU and CLSM results corroborate the manufacturer’s suggestion that repeated SDF application after one week will enhance its effectiveness in caries arrest. The 38% SDF solution contains high concentrations of silver (253,870 ppm) and fluoride (44,800 ppm) ions. Both the silver and fluoride ions released from the SDF appeared to have inhibited the cariogenic biofilms. Silver ions are bactericidal metal cations that inhibit biofilm formation [25] by inactivating and interfering with the bacterial synthesis of cellular polysaccharides through the inactivation of the glycosyltransferase enzymes responsible for the synthesis of soluble and insoluble glucan. Glucan not only contributes to the bulk of biofilms, but also plays an essential role in the sucrose-dependent adhesion of organisms to tooth surfaces [26]. Fluoride in high concentration also can inhibit biofilm formation [6]. Fluoride ions can bind to bacterial cell constituents and influence enzymes, such as enolase and proton-extruding adenosine triphosphatase (ATPase). The latter enzymes are considered to effectively inhibit the carbohydrate metabolism of acidogenic oral bacteria, as well as their sugar uptake [27].

Studies have shown that changes in the microhardness of dentin are directly related to its mineral content [19,28]. Therefore, measuring the microhardness is a reasonable, indirect method of examining the mineral content of dentin. Studies reported that SDF treatment increased the microhardness of carious dentin [29]. Another study reported that less soluble or virtually insoluble calcium fluoride, silver phosphate, and silver protein were formed and precipitated on the dentin surface when SDF was applied [30]. This formed an insoluble protective layer that decreased calcium and phosphorous loss from the carious lesions. In this study, precipitates containing high silver and phosphorus content (measured by EDX, data not shown) were observed occluding the tubule orifices after SDF application.

The organic dentin matrix mainly consists of collagen, which is the structural backbone holding the hydroxyapatite together [31]. Collagen fibers are exposed when hydroxyapatite is dissolved by acid. This profoundly increases the surface area and exposes more carbonyl groups. As a result, there is an increase in the FTIR signal on demineralized dentin [32]. In this study, the log [amide I: HPO_4_^2-^] of the control group increased over time, suggesting that more collagen I was exposed and less HPO_4_^2-^ remained in the dentin surface. Therefore, the demineralization of dentin progressed over time. In contrast, a lower log [amide I: HPO_4_^2-^] value was found in the test group, indicating that SDF could protect collagen I from further exposure, and that a regain of HPO_4_^2-^ might also occur [33]. Our recent laboratory study indicated that SDF has an inhibitory effect on matrix metalloproteinases (MMPs) [34]. MMPs play a crucial role in collagen breakdown in carious lesions [35]. SDF’s inhibition of MMP activities can protect against collagen degradation.

## Conclusions

According to the results of this study, 38% SDF arrests dental caries by reducing the demineralization process. It minimizes the loss of mineral content and slows down collagen I destruction. Furthermore, it contains high concentrations of silver and fluoride ions, which can inhibit the growth of multi-species cariogenic biofilms.

## Competing interest

The authors declared no potential conflicts of interest with respect to the authorship and/or publication of this article.

## Authors’ contribution

MLM carried out biofilm and hard tissue studies, and drafted the manuscript. CHC supervised the work, evaluated the results and corrected the manuscript for publication. QLL, ECML and LPS participated in the study design and coordination and helped to draft the manuscript. All authors read and approved the final manuscript.
